# The Changes in Optic Nerve after Orbital Decompression Surgery for Thyroid Eye Disease and Case Reports of Ischemic Optic Neuropathy

**DOI:** 10.1155/2022/4808194

**Published:** 2022-02-27

**Authors:** Yun Hsia, Chia-Chieh Hsiao, Yi-Hsuan Wei, I-Wen Lai, Chao-Wen Lin, Shu-Lang Liao

**Affiliations:** ^1^National Taiwan University Hospital Jin-Shan Branch, New Taipei City, Taiwan; ^2^Department of Ophthalmology, National Taiwan University Hospital, Taipei, Taiwan; ^3^College of Medicine, National Taiwan University, Taipei, Taiwan

## Abstract

**Purpose:**

To demonstrate the changes in the retinal nerve fiber layer (RNFL) after orbital decompression for thyroid eye disease (TED).

**Methods:**

We retrospectively enrolled 52 surgical TED patients, 30 nonsurgical TED patients, and 30 control subjects. Five surgical TED eyes with disc edema were excluded. The surgical TED patients were classified into the “dysthyroid optic neuropathy (DON)” group (16 eyes) and the “non-DON” group (83 eyes). Optical coherence tomography (OCT) and visual field (VF) examinations were performed preoperatively and 6 months later. The control subjects and nonsurgical TED patients received two OCT examinations at 6-month intervals. The postoperative changes in the RNFL thickness were compared between groups. Three cases with severe postoperative vision loss were presented additionally.

**Results:**

The changes in the RNFL thickness of the controls (0.5 ± 3.4 *μ*m) and the nonsurgical TED patients (0.3 ± 2.8 *μ*m) were significantly smaller than the surgical TED patients (*P* < 0.001). The DON group (−9.2 ± 9.2 *μ*m) had greater RNFL thickness reduction than the non-DON group (−3.9 ± 5.4 *μ*m) (*P* = 0.002). Bone removal decompression was associated with decreased RNFL in the non-DON (*P* = 0.025; *β* = −2.49) and DON (*P* = 0.042; *β* = −9.43) groups. Three cases who were hard to operate due to extensive fibrosis experienced severe vision loss postoperatively due to anterior ischemic optic neuropathy, posterior ischemic optic neuropathy, and posterior ciliary artery occlusion, respectively.

**Conclusions:**

TED patients experienced subclinical optic nerve injury and significant RNFL loss after the orbital decompression surgery. Aggressive manipulation during decompression surgery may lead to dreadful vision loss. Tailored surgical plans and delicate manipulation are warranted.

## 1. Introduction

Orbital decompression is a well-recognized and effective surgical procedure for thyroid eye disease (TED) [1–3]. It can be performed in inactive TED for disfiguring exophthalmos or in the active phase for dysthyroid optic neuropathy (DON) refractory to medical treatment [2–5]. Different surgical approaches are tailored to the individual patient based on the extent of proptosis, preoperative diplopia, and the presence of DON [3, 4].

Vision loss is a very rare but devastating complication after orbital surgery [6–11]. The prevalence of vision loss after TED decompression surgery ranges from 0.09 to 0.52% [8, 11, 12]. The mechanical injury to the optic nerve or ischemia caused by compression from swelling tissue and hematoma, hypotension during general anesthesia, vasospasm, and arterial occlusion leads to vision loss [11]. In the present study, we reported the clinical manifestations, possible etiologies, and treatment of patients with severe vision loss after orbital decompression surgeries.

The thickness of the retinal nerve fiber layer (RNFL) decreased after orbital decompression surgery in eyes with TED and DON. This could be attributed to the mixture of resolved disc swelling and chronic optic nerve atrophy [13, 14]. However, no study had investigated the RNFL changes in TED eyes without DON. We postulated that orbital decompression surgery per se might cause minor and subclinical injury to the optic nerve. Therefore, we evaluated the changes in parameters of optical coherence tomography (OCT) and visual field (VF) tests in TED patients with and without DON after orbital decompression surgery.

## 2. Methods

We retrospectively enrolled patients with TED receiving orbital decompression surgery (the surgical TED group) between December 2017 and November 2020 in National Taiwan University Hospital. The diagnosis of TED was based on the consensus of the European Group on Graves' Orbitopathy (EUGOGO) [15]. All patients underwent comprehensive examinations, including the slit-lamp biomicroscopy, the measurement of best-corrected visual acuity (BCVA) and intraocular pressure (CT-80 Non-Contact Computerized Tonometer; Topcon Corp, Tokyo, Japan), Hertel exophthalmometry, color fundus photography, and orbital computerized tomography (CT). Patients with optic neuropathy other than DON, retinal diseases, history of intraocular surgery other than cataract surgery, or ocular trauma were excluded. Patients with disc swelling on indirect ophthalmoscope or color fundus photography were excluded. The diagnosis of DON was made if the patient had decreased visual acuity (logarithm of the minimal angle of resolution [LogMAR] visual acuity > 0.2) due to optic neuropathy and the following presentations: impaired color vision, and/or relative afferent pupillary defect (RAPD), and/or prolonged latency and reduced amplitude of visual evoked potentials test, and/or VF defect, and apical crowding on orbital imaging [16, 17]. The treatment algorithms for the TED were described in our previous publication [4]. The most common surgical indication in the non-DON group is to correct the disfiguring exophthalmos. The surgery would be arranged when the patient had stable thyroid function and inactive TED (clinical activity score [CAS] < 3) for 3-6 months. A seven-item CAS was used, including spontaneous retrobulbar pain, pain on upward or downward gaze, redness of eyelids, redness of conjunctiva, swelling of caruncle or plica, swelling of eyelids, and chemosis. Patients presented with active TED were treated with systemic steroids or other immunosuppressants before the surgery. If the patient had vision-threatening conditions, such as optic neuropathy and exposure keratopathy, the surgery would be arranged after the active inflammation was at least partially controlled by systemic treatments.

Medical charts were retrospectively reviewed, and the relevant data at preoperative evaluation and postoperative 6 months were recorded. TED patients who did not receive orbital decompression (the nonsurgical TED group) and control subjects without optic neuropathy and retinal diseases were enrolled as controls, respectively. Institutional Review Board/Ethics Committee approval was obtained, and the research adhered to the tenets of the Declaration of Helsinki. The informed consent was waived.

### 2.1. Spectral-Domain OCT

Optic nerve head scanning was performed using spectral-domain OCT (Cirrus HD-OCT 4000, Carl Zeiss Meditec Inc., Dublin, CA, USA). The average RNFL thickness, RNFL thickness at each quadrant, rim area, disc area, cup-to-disc ratio, and cup volume were recorded. Images with signal strength < 7 or motion artifact were excluded. Only patients with OCT taken preoperatively and at postoperative 6 months would be included for analysis. The average RNFL thickness of nonsurgical TED patients and control subjects with two available OCT scans at a 6-month-interval were recorded.

### 2.2. VF Test

The VF test (Humphrey visual field analyser, Carl Zeiss Meditec, Dublin, CA, USA) was performed using the 24–2 SITA-fast program. Reliable visual field test result was defined as false positive < 33%, false negative < 33%, and fixation loss < 20%. Mean deviation (MD), pattern standard deviation (PSD), and visual field index (VFI) were recorded.

### 2.3. Statistical Analysis

All statistical analyses were performed using *R* (version 4.0.3; R Foundation for statistical computing, Vienna, Austria). The age and sex between TED patients and controls were compared using the analysis of variance and chi-squared test, respectively. The generalized linear model and generalized estimating equations (GEE) were used to compare the clinical features between TED patients and to compare the clinical features before and after the orbital decompression surgery. To analyze the factors associated with the RNFL changes after the orbital decompression surgery, univariable linear regression with GEE model was performed. Variables with *P* value <0.1 were included in the multivariable model. *P* value <0.05 was considered statistically significant.

## 3. Results

We enrolled 52 patients (104 eyes) in the surgical TED group, 30 patients (60 eyes) in the nonsurgical TED group, and 30 control subjects (60 eyes). Five TED eyes in the surgical group had disc swelling noted on fundus examination and were excluded due to possible interference on OCT interpretation. In the surgical TED group, 16 eyes from 11 patients had DON (the DON group), while 83 eyes from 45 patients were classified into the non-DON group.  Table 1 shows the baseline clinical features of TED patients and control subjects. There was no difference in sex and age between TED patients and control subjects. To compare the nonsurgical and surgical TED group, the surgical group had more significant proptosis (*P* < 0.001), higher intraocular pressure (*P* < 0.001), worse MD (*P* < 0.001), and higher CAS (*P* < 0.001). In the surgical TED group, the DON group was older (*P* = 0.012) and had male predominance (82%) (*P* = 0.020). DON patients tended to receive bone removal orbital decompression (BROD) (*P* = 0.010) and had worse visual acuity (*P* = 0.005) and MD (*P* < 0.001). Twenty-five (55.6%) non-DON patients and 6 DON (54.5%) patients received systemic steroid treatment before the surgery, respectively (*P* = 0.999). The number of patients with abnormal thyroid function tests was not significantly different between the non-DON and DON groups (*P* = 0.999).

**Figure 1:**
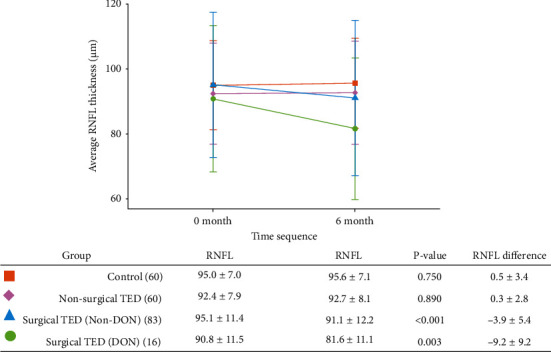
The changes in the thickness of the retinal nerve fiber layer (RNFL) of patients with thyroid eye disease (TED) and healthy controls. There is no significant difference in RNFL thickness between the four groups at baseline. Surgical TED patients have a significant RNFL loss after the orbital decompression surgery, while the control subjects and nonsurgical TED patients do not have RNFL changes between two examinations of the optical coherence tomography.

Table 2 demonstrates the comparison of clinical features before and after orbital decompression surgery. After the surgery, proptosis and intraocular pressure decreased significantly in both groups. Both the non-DON (*P* < 0.001) and the DON (*P* = 0.003) group had a significant reduction of average RNFL thickness. The average cup-to-disc ratio and cup volume increased in both groups. The parameters of the VF test did not change in the non-DON group, while the MD and PSD improved in the DON group.  Figure 1 shows the changes in average RNFL thickness of the surgical and nonsurgical TED patients and the control subjects. The baseline RNFL thickness was not different between the DON and non-DON groups (*P* = 0.200). The changes in RNFL thickness of the control subjects (0.5 ± 3.4 *μ*m) and the nonsurgical TED patients (0.3 ± 2.8 *μ*m) were significantly smaller than the surgical TED patients (*P* < 0.001 in both DON and non-DON groups). The DON group (−9.2 ± 9.2 *μ*m) had greater RNFL thickness reduction than the non-DON group (−3.9 ± 5.4 *μ*m) (*P* = 0.002).

Table 3 reveals factors associated with the change in RNFL after orbital decompression surgery in the univariable model. In the non-DON group, the multivariable linear regression showed that BROD (*P* = 0.025; *β* = −2.49), baseline MD (*P* < 0.001; *β* = 0.64), and age (*P* = 0.040; *β* = −0.12) were associated with the change in average RNFL thickness after surgery. In the DON group, the multivariable linear regression showed that BROD (*P* = 0.042; *β* = −9.43) and the baseline RNFL thickness of nasal quadrant of RNFL (*P* = 0.002; *β* = −0.54) were associated with the change in average RNFL thickness after surgery. Receiving BROD was associated with decreased RNFL in both groups.

Despite extremely low prevalence, severe vision loss could occur after orbital decompression surgery for TED. We identified three patients and described the detailed clinical information in the following sections. Their data was not included in the analysis mentioned above.

### 3.1. Case 1

**Figure 2:**
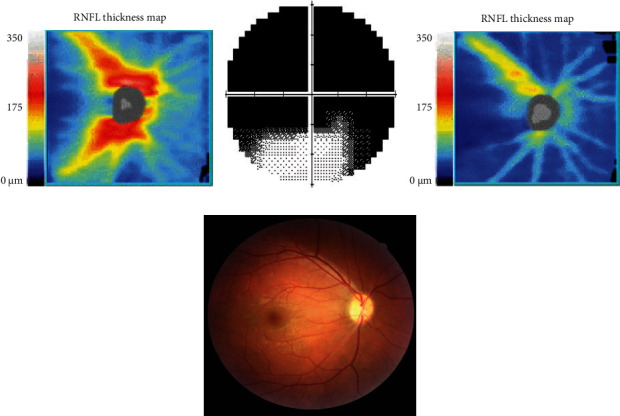
Clinical presentations of a patient with thyroid eye disease experience severe vision loss after the decompression surgery (case 1). The patient reports visual field defect after an uneventful fat-removal orbital decompression. (a) Initially, the optical coherence tomography scan shows intact retinal nerve fiber layer (RNFL), and the average thickness is 96 *μ*m. (b) However, the visual field test shows upper visual field defect with the mean deviation -27.17 dB. (c) Diffuse loss of inferior RNFL is noted, and the average thickness is 61 *μ*m three months after the surgery. (d) Color fundus photography reveals that the optic disc becomes pallor. The clinical presentations are compatible with posterior ischemic optic neuropathy.

A male patient in his 20s had proptosis with 22 mm for the right eye and 21 mm for the left eye measured by Hertel exophthalmometry. There was no evidence of DON, and his BCVA was 20/20 in both eyes. Subconjunctival FROD was performed, and 5.6 ml and 5.1 ml of orbital fat were removed from his right and left eye, respectively. However, he complained VF defect in the right eye two days after the surgery. Although his BCVA was unchanged, he had a dilated pupil, a positive RAPD sign, and impaired color vision. Indirect ophthalmoscopy showed a pinkish disc with a clear margin. OCT scan showed normal and symmetric RNFL (Figure 2(a)). However, the VF test revealed severe upper defect (MD, -27.17 dB) (Figure 2(b)). Magnetic resonance imaging (MRI) scan showed no intraorbital hematoma or enhancement along the optic nerve. The tentative diagnosis was posterior ischemic optic neuropathy (PION). Intravenous steroid pulse therapy was given for three days (250 mg four times per day). However, the OCT scan showed inferior RNFL loss (Figure 2(c)), the right optic disc became pallor (Figure 2(d)), and his BCVA declined to 20/25 three months later.

### 3.2. Case 2

**Figure 3:**
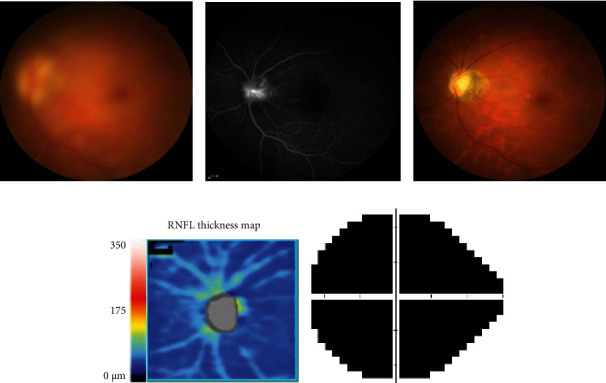
Clinical presentations of a patient with thyroid eye disease experience severe vision loss after the decompression surgery (case 2). The patient reports decreased vision to number of digits after an uneventful fat-removal orbital decompression. (a) Color fundus photography shows significant disc edema. The image is blurry due to corneal edema. (b) Fluorescein angiography shows disc leakage. The clinical presentations are compatible with anterior ischemic optic neuropathy. (c) Despite the prompt treatment, the optic disc becomes pallor. (d) The optical coherence tomography scan shows diffuse retinal nerve fiber layer loss, and the average thickness was 54 *μ*m. (e) The mean deviation of visual field test is -33.34 dB.

A female patient in her 40s had proptosis with 20 mm for the right eye and 22 mm for the left eye. There was no evidence of DON, and her BCVA was 20/30 in both eyes. Subconjunctival FROD was performed, and 4.2 ml and 5.5 ml of orbital fat were removed from her right and left eye, respectively. However, prominent hemorrhagic chemosis, extraocular muscle movement limitation in horizontal gaze, positive RAPD sign, and decreased vision to finger counting in the left eye were noted on postoperative day 1. Indirect ophthalmoscopy showed marked disc swelling (Figure 3(a)). Fluorescein angiography demonstrated disc leakage (Figure 3(b)). Orbital CT scan revealed some hematoma in the left orbit without optic nerve compression. The tentative diagnosis was anterior ischemic optic neuropathy (AION). Emergent hematoma evacuation was performed due to rapid worsening of visual acuity to hand motion. Intravenous steroid pulse therapy (250 mg four times per day) was given for three days. Unfortunately, the visual improvement was limited, and her BCVA was only finger counting at 10 cm 6 months later. The disc became pallor, and the OCT scan showed diffuse RNFL loss. The visual field defect was significant (Figures 3(c)–3(e)).

### 3.3. Case 3

**Figure 4:**
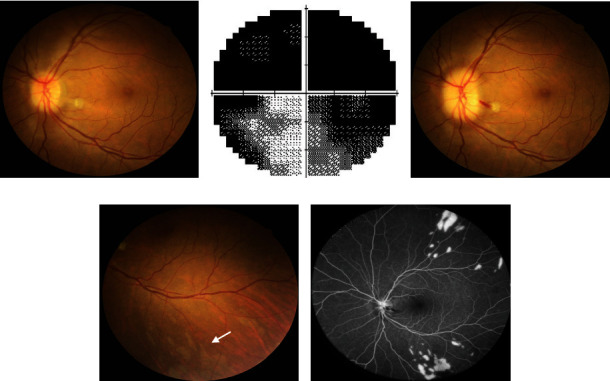
Clinical presentations of a patient with thyroid eye disease experience severe vision loss after the decompression surgery (case 3). The third patient reports progressive loss of vision after an uneventful fat-removal orbital decompression. (a) Initially, color fundus photography showed disc edema. (b) Visual field test shows a superior defect, and the mean deviation is -25.06 dB. Two days later, the vision decreases to the hand motion level. (c) Color fundus photography shows the progression of disc edema. There are also a cotton-wool spot and disc hemorrhage. (d) There are also some triangular, well-defined, patchy choroidal ischemic lesions (arrow). (e) Fluorescein angiography reveals marked delayed choroidal perfusion and late staining of the patchy lesions. The clinical presentations are compatible with posterior ciliary artery occlusion.

A female patient in her 50s had proptosis with 21.5 mm for both eyes. There was no evidence of DON, and her BCVA was 20/20 in both eyes. Subconjunctival FROD was performed, and 5 ml and 4.8 ml of orbital fat were removed from her right and left eye, respectively. However, on postoperative day 1, she reported decreased visual acuity (20/30) and VF defect. On examinations, there were a mild RAPD sign, decreased color vision, and inferior optic disc swelling in the left eye (Figure 4(a)). The VF test showed superior defect, and the MD was -25.06 dB (Figure 4(b)). Emergent orbital CT scan and MRI showed no intraorbital hematoma, optic nerve avulsion, or definite optic neuropathy. Two days after the operation, the vision suddenly decreased to hand motion at 50 cm. Indirect ophthalmoscopy showed marked optic disc swelling. Color fundus photography showed triangular, well-defined, patchy infarcts lying deep to the retina. Fluorescence angiography revealed delayed choroidal perfusion and late staining of the patchy lesions in the left eye (Figures 4(c)–4(e)). The tentative diagnosis was posterior ciliary artery occlusion. Intravenous pulse steroid therapy (250 mg four times per day), subcutaneous enoxaparin (30 mg twice per day), and oral ginkgocentrate and mecobalamin were given for five days. Despite the prompt management, the left eye had no light perception three days later.

## 4. Discussion

Orbital decompression surgery had a close approximation to numerous neurovascular structures. Damage to those structures could lead to devastating complications. We found that RNFL thickness decreased significantly in TED patients after orbital decompression surgery. In contrast, TED patients who did not receive orbital decompression had no decline in RNFL thickness. Patients with DON had a more dramatic decrease in RNFL thickness, which may be associated with the additional effect of resolving disc edema or chronic optic nerve atrophy. Moreover, patients who received BROD were more susceptible to RNFL loss than those receiving FROD. However, the RNFL change after orbital decompression surgery did not cause functional deterioration. Despite the extremely low prevalence of postoperative vision loss, we identified three patients who suffered from significant vision loss after orbital decompression surgery due to AION, PION, and posterior ciliary artery occlusion, respectively.

In the present study, we would like to know whether the orbital decompression surgery may damage the optic nerve. The RNFL thickness varies significantly between TED patients with different severities [18]. Patients with mild TED had comparable RNFL thickness to controls, while those with moderate-to-severe TED have lower value of RNFL thickness due to subclinical optic nerve damage [18]. Orbital tissue swelling and elevated intraorbital pressure may lead to hypoxia and ischemia of the optic nerve head. This was supported by the fact that peripapillary vessel density was lower in eyes with active TED [19, 20]. Patients with acute DON had increased RNFL thickness due to disrupted axoplasmic flow and optic disc swelling [20], while patients with chronic DON had thinner RNFL due to optic nerve atrophy [21]. Therefore, we classified TED patients into the DON and the non-DON groups to clarify the changes of RNFL thickness after orbital decompression surgery.

We found that the baseline RNFL thickness in both DON and non-DON groups was comparable to the control subjects. The RNFL thickness in the DON group may reflect the mixed effects of disc swelling and optic nerve damage, while there was no evidence of structural change in the non-DON group. In order to eliminate the possible test-retest variability of OCT scans, we compared the RNFL changes of the TED eyes to the two measurements of control subjects separated by six months. After the surgery, TED eyes had a significant RNFL deterioration compared to the controls. The DON and non-DON groups had a 9 and 4 *μ*m RNFL decrease, respectively. A decrease of RNFL thickness by 4 *μ*m measured with Cirrus OCT should be considered a true deterioration [22, 23]. In order to clarify the possibility of RNFL deterioration due to the disease nature of TED, we compared the surgical TED patients to the TED patients who did not receive orbital decompression. The nonsurgical TED patients did not show significant RNFL deterioration during the follow-up. Since the nonsurgical TED patients did not need surgical intervention, it was reasonable that they had less significant proptosis, lower intraocular pressure, and CAS than the patients in the surgical TED group. However, the proptosis was corrected, and intraocular pressure decreased immediately after the orbital decompression. Therefore, the postoperative RNFL loss in the surgical TED group may be mainly attributed to the surgical traction to the optic nerve and its supplying blood vessels and the compression from postoperative tissue swelling. The resolving disc edema and chronic optic nerve atrophy may explain that the DON group had more RNFL loss [13, 14]. However, the role of resolving disc edema is less critical in our cohort since we excluded cases with disc swelling. The vulnerable optic nerve in the DON group may be more susceptible to the RNFL loss related to decompression surgery. Moreover, we found that BROD was associated with a more significant decrease in RNFL thickness than the FROD in both DON and non-DON groups. BROD had a higher complication rate due to the alteration of normal orbit anatomy [1, 24]. In the non-DON group, older patients and those with preoperative VF defects had a smaller neuronal reserve and were more vulnerable to the damage associated with decompression surgery. Worth to be mentioned, the systemic steroid treatment before the orbital decompression was associated with less RNFL deterioration in the DON group. It might be possible that those who did not take steroids still had subclinical optic nerve swelling. Therefore, they exhibited more significant RNFL reduction after the operation due to the mixed effect of the resolution of edema and true RNFL loss. However, this association became nonsignificant in the multivariable regression analysis.

Vision loss had been reported as a rare complication after various orbital surgeries, including tumor excision, posttraumatic reconstruction, and decompression for TED [9, 11]. Among these orbital surgeries, orbital decompression for TED had the lowest risk of vision loss [9, 11]. One patient receiving endoscopic BROD had vision loss caused by ischemic optic neuropathy one week later [7]. Other studies regarding the outcomes of orbital decompression surgery also reported very few cases with postoperative vision loss [8, 12, 25]. The possible etiologies of vision loss included surgical manipulation, thermal and electrical injury, compression from bony fragments, vasospasm due to inflammation, and elevated intraorbital pressure due to tissue swelling, retrobulbar hemorrhage, and tight pressure patching [10, 11]. Ischemic events included AION, PION, posterior ciliary artery occlusion, central retinal artery occlusion, or ophthalmic artery occlusion could occur [10, 11].

In the present study, three cases with severe vision loss after FROD had different underlying etiologies. The first patient had an episode of PION. Hypotension during general anesthesia [26] was not noted. The surgical traction may damage the pial plexus directly or cause vasospasm and compromise the blood supply of the intraorbital optic nerve [11]. Marked tissue swelling may also increase intraorbital pressure and reduce blood perfusion. Since we removed the inferior orbital fat and possibly damaged the inferior portion of optic nerve, the patient had a corresponding superior VF defect. The optic disc appeared normal at the acute phase but became pallor 4-6 weeks later [26]. The second patient had AION. Although peribulbar hematoma was identified on imaging, there was no evidence of direct compression on the optic nerve. The increased intraorbital pressure and the postoperative inflammation may contribute to the optic nerve head ischemia. Emergent surgical evacuation of hematoma had a limited effect on the visual prognosis of this case. The third patient had marked and progressive disc swelling. Moreover, delayed choroidal perfusion and ischemia of the outer retina were noted on fluorescein angiography. Infarction of the short posterior ciliary artery led to ischemia of the optic nerve head, choroid, and outer retina [27]. Acute choroidal ischemic lesions are localized and discrete whitish patches and distribute along the territory of the occluded branch of short posterior ciliary artery. Later, they evolve into depigmented lesions [27]. This patient ended up with no light perception due to extensive ischemia. Intravenous steroid pulse therapy had been given in these cases but did not enhance the visual improvement. Subcutaneous enoxaparin injection had been advocated in cases with posterior ciliary artery occlusion due to filler injection [28]; however, the effect was also insubstantial in our patient. All these three patients were relatively hard to operate due to extensive fibrosis and adhesion. Aggressive surgical manipulations may predispose to these complications. Therefore, a more conservative surgical plan should be adopted in cases with limited surgical fields to avoid dreadful vision loss.

This study had several limitations. Since we aimed to eliminate the effect of resolving disc edema on postoperative RNFL change, we excluded patients with disc edema. Therefore, the number of DON patients was small, and the data may be less representative for this population. Patients who had ocular hypertension tended to have OCT scans more frequently and were more likely to be included. However, we excluded patients with glaucomatous optic neuropathy, and the intraocular pressure decreased significantly after orbital decompression. Therefore, the intraocular pressure was unlikely to cause the RNFL loss. Additionally, we only observed the RNFL changes 6 months after the surgery. Longer follow-up was necessary to decide whether this RNFL deterioration was an isolated event related to orbital decompression surgery or was progressive due to the chronic optic nerve atrophy. Lastly, different types of orbital decompression, such as medial or lateral wall decompression or other orbital surgeries, including orbital fracture repair and orbital tumor excision, may have different effects on the RNFL. More comprehensive analysis is needed to clarify the impact on RNFL caused by a specific type of orbital surgery.

In conclusion, we found that TED patients had a significant RNFL decrease after the orbital decompression surgery, especially those with DON. BROD was associated with a greater RNFL decrease than FROD. Additionally, we reported three cases with severe vision loss after FROD for TED, presented as AION, PION, and posterior ciliary artery occlusion, respectively. Aggressive surgical manipulation due to extensive adhesion and fibrosis may lead to the complications. Therefore, staying mindful of potential complications and optic nerve damage is imperative. Tailored surgical plan, delicate intraoperative manipulation, and appropriate postoperative follow-up are warranted in TED patients receiving orbital decompression surgery.

## Figures and Tables

**Table 1:** Baseline demographics and clinical features of patients with thyroid eye disease and controls.

	Between-group comparison				Subgroup analysis: Surgical TED^a^		
	Control	Nonsurgical TED	Surgical TED	*P* value	Non-DON	DON	*P* value
Patients (*n*)	30	30	52		45	11	
Eyes (*n*)	60	60	99		83	16	
Age (years)	51.7 ± 5.8	56.7 ± 10.2	53.8 ± 10.6	0.057	52.6 ± 10.6	60.1 ± 8.5	**0.012**
Sex (male)	13 (43.3)	9 (30)	23 (43.4)	0.439	17 (37.8)	9 (81.8)	**0.020**
Hertel exophthalmolmetry (mm)		18.2 ± 3.4	22.1 ± 2.3	**<0.001**	22.0 ± 2.2	22.5 ± 3.0	0.560
BROD (%)		NA	49 (49.5)		36 (43.4)	13 (81.3)	**0.010**
FROD (%)		NA	50 (50.5)		47 (56.6)	3 (18.7)	**0.010**
Intraocular pressure (mmHg)		16.3 ± 2.5	22.3 ± 5.4	**<0.001**	21.8 ± 5.2	24.6 ± 6.0	0.130
LogMAR visual acuity		0.05 ± 0.12	0.10 ± 0.23	0.074	0.06 ± 0.11	0.38 ± 0.44	**0.005**
EOM limitation (%)		33 (55)	66 (66.7)	0.220	52 (62.7)	14 (87.5)	0.100
EOM enlargement (%)		47 (78.3)	77 (77.8)	0.987	61 (73.5)	16 (100)	**0.040**
Mean deviation (dB)		−1.24 ± 1.44	−3.74 ± 4.55	**<0.001**	−2.74 ± 3.63	−8.95 ± 5.37	**<0.001**
Abnormal thyroid function (%)		8 (26.7)	20 (37.7)	0.400	16 (35.6)	4 (36.4)	0.999
Preoperative clinical activity score		1 (0-2)	1 (0-7)	**<0.001**	1 (0-7)	2 (0-4)	0.310

^a^Four patients had one eye in the non-DON group and the other eye in the DON group. BROD: bone removal orbital decompression; DON: dysthyroid optic neuropathy; EOM: extraocular muscle; FROD: fat-removal orbital decompression; LogMAR: logarithm of the minimum angle of resolution; NA: not applicable; TED: thyroid eye disease; mean ± standard deviation; median: range; number: percent. Significant *P* values are shown in bold.

**Table 2:** Clinical characteristics before and after orbital decompression surgery.

	Non-DON (83 eyes)			DON (16 eyes)		
	Preoperative	Postoperative	*P* value	Preoperative	Postoperative	*P* value
Average RNFL (*μ*m)	95.1 ± 11.4	91.1 ± 12.2	**<0.001**	90.8 ± 11.5	81.6 ± 11.1	**0.003**
Rim area (mm^2^)	1.20 ± 0.28	1.18 ± 0.32	0.170	1.16 ± 0.25	0.99 ± 0.20	**<0.001**
Average cup-to-disc ratio	0.56 ± 0.15	0.60 ± 0.15	**0.002**	0.53 ± 0.19	0.62 ± 0.12	**0.008**
Vertical cup-to disc ratio	0.52 ± 0.16	0.56 ± 0.16	**0.002**	0.48 ± 0.20	0.57 ± 0.16	0.110
Cup volume (mm^3^)	0.246 ± 0.227	0.280 ± 0.243	**<0.001**	0.215 ± 0.209	0.268 ± 0.225	**<0.001**
RNFL of superior quadrant (*μ*m)	115.0 ± 19.4	109.0 ± 22.1	**<0.001**	110.1 ± 22.4	97.4 ± 19.5	**0.001**
RNFL of temporal quadrant (*μ*m)	80.6 ± 15.5	78.7 ± 16.1	0.170	74.4 ± 13.0	65.8 ± 14.7	**0.018**
RNFL of inferior quadrant (*μ*m)	117.0 ± 22.1	110.0 ± 22.1	**<0.001**	108.8 ± 19.7	100.4 ± 20.4	**0.003**
RNFL of nasal quadrant (*μ*m)	67.5 ± 12.2	66.0 ± 12.5	**0.009**	70.2 ± 11.7	66.7 ± 8.6	0.100
Mean deviation (dB)	−2.74 ± 3.63	−2.38 ± 3.82	0.160	−8.95 ± 5.37	−5.25 ± 4.46	**0.047**
Pattern standard deviation (dB)	2.56 ± 2.32	2.42 ± 2.37	0.510	5.42 ± 3.37	2.97 ± 2.19	**0.016**
Visual field index (%)	95.3 ± 8.2	95.1 ± 9.7	0.800	80.6 ± 13.7	90.8 ± 13.8	0.058
Hertel exophthalmometry (mm)	22.0 ± 2.2	17.4 ± 1.7	**<0.001**	22.5 ± 3.0	17.7 ± 2.5	**<0.001**
LogMAR visual acuity	0.06 ± 0.11	0.07 ± 0.15	0.450	0.38 ± 0.44	0.26 ± 0.42	0.053
Intraocular pressure (mmHg)	21.8 ± 5.2	17.1 ± 3.0	**<0.001**	24.6 ± 6.0	16.7 ± 4.0	**<0.001**

DON: dysthyroid optic neuropathy; EOM: extraocular muscle; LogMAR: logarithm of the minimum angle of resolution; TED: thyroid eye disease. Significant *P* values are shown in bold.

**Table 3:** The univariable analysis of factors associated with RNFL deterioration after decompression.

	Non-DON		DON	
	*β*-Coefficients (95% CI)	*P* value	*β*-Coefficients (95% CI)	*P* value
Age (years)	-0.15 (-0.29–-0.01)	**0.035**	-0.04 (-0.57–0.50)	0.898
Male sex	-1.68 (-4.75–1.40)	0.285	6.57 (0.71–12.40)	**0.028**
Intraocular pressure (mmHg)	0.04 (-0.13–0.20)	0.646	0.22 (-0.54–0.98)	0.563
LogMAR visual acuity	-13.0 (-23.00–-3.04)	**0.011**	-3.22 (-11.60–5.13)	0.450
Mean deviation (dB)	0.58 (0.29–0.87)	**<0.001**	-0.01 (-0.72–0.71)	0.989
Average RNFL (*μ*m)	-0.09 (-0.24–0.06)	0.222	-0.58 (-0.87–-0.29)	**<0.001**
RNFL of superior quadrant (*μ*m)	-0.02 (-0.09–0.05)	0.506	-0.17 (-0.31–0.03)	**0.014**
RNFL of temporal quadrant (*μ*m)	-0.05 (-0.12–0.02)	0.168	-0.27 (-0.67–0.13)	0.186
RNFL of inferior quadrant (*μ*m)	-0.03 (-0.11–0.05)	0.474	-0.18 (-0.38–0.03)	0.089
RNFL of nasal quadrant (*μ*m)	-0.07 (-0.16–0.03)	0.167	-0.51 (-0.77–-0.25)	**<0.001**
Hertel exophthalmometry (mm)	0.05 (-0.48–0.59)	0.855	4.82 (2.28–7.36)	**<0.001**
BROD	-2.48 (-4.79–-0.18)	**0.035**	-7.95 (-15.40–-0.54)	**0.036**
Diplopia	1.36 (-1.41–4.13)	0.336	4.90 (-5.30–15.10)	0.347
EOM enlargement	-0.93 (-3.36–1.50)	0.453	Not available^∗^	Not available^∗^
Clinical activity score	0.20 (-0.78–1.17)	0.690	4.35 (2.85–5.85)	**<0.001**
Systemic steroid treatment	-2.12 (-4.78–0.53)	0.117	10.3 (3.61–17.00)	**<0.001**

BROD: bone removal orbital decompression; DON: dysthyroid optic neuropathy; LogMAR: logarithm of the minimum angle of resolution. The value of change in RNFL equals the postoperative average RNFL thickness minus the preoperative average RNFL thickness. ^∗^The results are not available since all patients in the DON group had EOM enlargement. Significant *P* values are shown in bold.

## Data Availability

The data presented in this study are available on request from the corresponding author. The data are not publicly available due to ethical and privacy concerns.
